# Finite Element Analysis of the Effects of Different Shapes of Adult Cranial Sutures on Their Mechanical Behavior

**DOI:** 10.3390/bioengineering12030318

**Published:** 2025-03-19

**Authors:** Han Yang, Shiguo Yuan, Yuan Yan, Li Zhou, Chao Zheng, Yikai Li, Junhua Li

**Affiliations:** 1 School of Traditional Chinese Medicine, Southern Medical University, Guangzhou 510515, China; 2 Guangdong Provincial Hospital of Chinese Medicine, Hainan Hospital, Guangzhou University of Chinese Medicine, Haikou 570311, China; 3 Hainan Traditional Chinese Medicine Hospital, Hainan Medical University, Haikou 570311, China; 4 The Third Affiliated Hospital, Sun Yat-sen University, Guangzhou 510700, China; 5 The Third Affiliated Hospital, Southern Medical University, Guangzhou 510630, China; 6 The Affiliated Traditional Chinese Medicine Hospital, Guangzhou Medical University, Guangzhou 510130, China

**Keywords:** finite element method, cranial suture, mechanical behavior, impact and tensile loads, craniosacral therapy

## Abstract

Cranial sutures play critical roles in load distribution and neuroprotection, with their biomechanical performance intimately linked to morphological complexity. The purpose of this study was to investigate the effect of different morphologies of cranial sutures on their biomechanical behavior. Based on the different morphologies of the cranial sutures, six groups of finite element models (closed, straight, sine wave, tight sinusoidal wave, layered sinusoidal wave, and layered sinusoidal wave + sutural bone) of the bone–suture–bone composite structures that ranged from simple to complex were constructed. Each model was subjected to 50 kPa impact and 98 N bilateral tensile loads to evaluate von Mises stress and total deformation variations across all groups under combined loading conditions. Key findings reveal that morphological complexity directly governs stress dynamics and mechanical adaptation; layered sinusoidal configurations delayed peak stress by 19–36% and generated elevated von Mises stresses compared to closed sutures, with stress concentrations correlating with interfacial roughness. Under impact, sutures exhibited localized energy dissipation (<0.2 μm deformation), while tensile loading induced uniform displacements (≤11 μm) across all morphologies (*p* > 0.05), underscoring their dual roles in localized energy absorption and global strain redistribution. Craniosacral therapy relevant forces produced sub-micron deformations far below pathological thresholds (≥1 mm), which implies the biomechanical safety of recommended therapeutic force. Staggered suture–bone in open sutures (31.93% closure rate) enhances shear resistance, whereas closed sutures prioritize rigidity. The findings provide mechanistic explanations for suture pathological vulnerability and clinical intervention limitations, offering a quantitative foundation for future research on cranial biomechanics and therapeutic innovation.

## 1. Introduction

Cranial sutures, as fibrous articulations linking cranial bones, serve critical biomechanical functions in developmental morphogenesis and impact energy dissipation and neurosensory protection, with translational relevance for traumatic injury mechanisms [1,2]. Extensive in vivo studies using micro-CT imaging, histomorphometry, and mechanical testing have demonstrated that cranial sutures function as dynamic load-bearing structures whose mechanical properties are intimately linked to their geometric complexity [3,4]. Computational modeling approaches, including finite element analysis (FEA), have further advanced our understanding by revealing how sutural geometry influences stress distribution patterns and energy absorption capacity during impact loading [3,4,5,6].

Despite these advances, contemporary computational models remain constrained by oversimplified geometric representations. Previous FE studies by Rayfield [7], Jasinoski et al. [8], and Maloul et al. [9] reported the effects of the material properties of cranial sutures (isotropic, transverse isotropic, and viscoelastic), geometric morphology, and the loading direction (perpendicular or parallel to the cranial suture) on the mechanical behavior of the bone–suture–bone composite structure. However, these investigations primarily employed idealized sinusoidal geometries, neglecting the morphological diversity observed in vivo. According to our findings in previous anatomical studies [10,11], cranial sutures exhibit diverse morphologies and demonstrate heterogeneous mechanical responses. Cranial sutures can be characterized as closed lines, straight lines, regular waveforms (e.g., sine waves), or complex shapes incorporating interstitial bone within sutural regions [10,11]. This morphological variation implies that geometric parameters (e.g., fractal complexity) may play critical roles in modulating mechanical behavior [1,2].

The controversy surrounding craniosacral therapy (CST) provides a practical context for this research. Originating in the US and gaining popularity in Asia [12], CST posits that cranial sutures can be manipulated with forces between 5 and 10 g [13]. While some clinical studies report symptomatic improvements [12], the central hypothesis that sutural mobility can be induced through manual manipulation remains unsubstantiated by biomechanical evidence. This study bridges morphometric analysis of cranial sutures with parametric finite element modeling to investigate the biomechanical behavior of six distinct suture morphologies under impact loading (simulating external impact forces) and tensile loading (mimicking manual tension). These findings may provide a framework for understanding suture mechanics across physiological functions and pathological deformations in order to offer a quantitative reference for interpreting cranial suture mechanics under clinically relevant loading conditions and anatomically derived morphometric descriptors.

## 2. Materials and Methods

### 2.1. FE Modeling of Cranial Sutures

**Figure 1:**
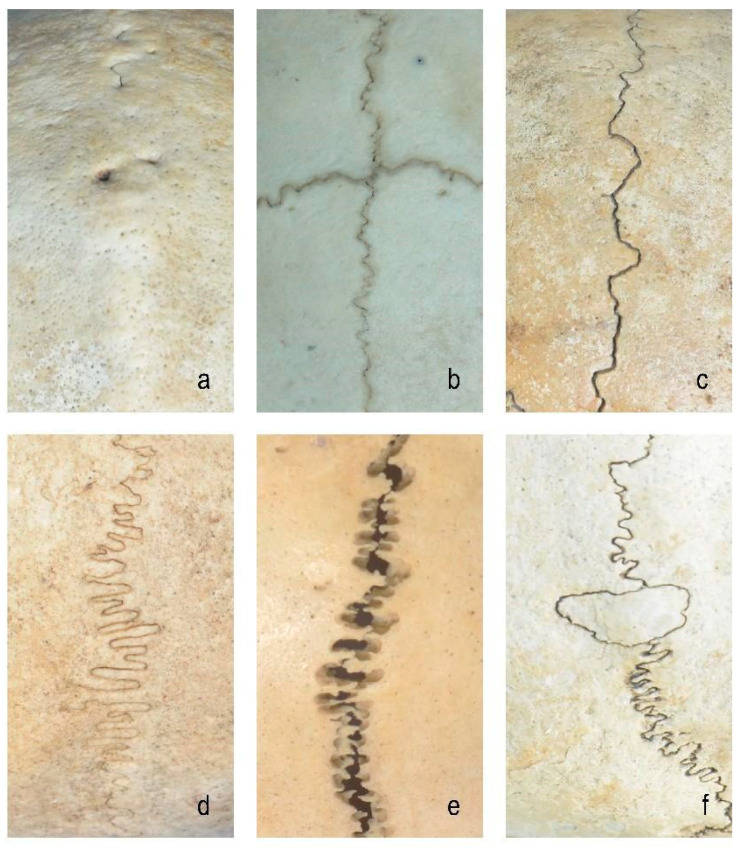
The six groups of anatomical morphology of cranial sutures: (**a**) closed group; (**b**) straight suture group; (**c**) sine wave group; (**d**) tight sinusoidal wave group; (**e**) layered sinusoidal wave group; (**f**) layered sinusoidal wave + sutural bone group.

**Figure 2:**
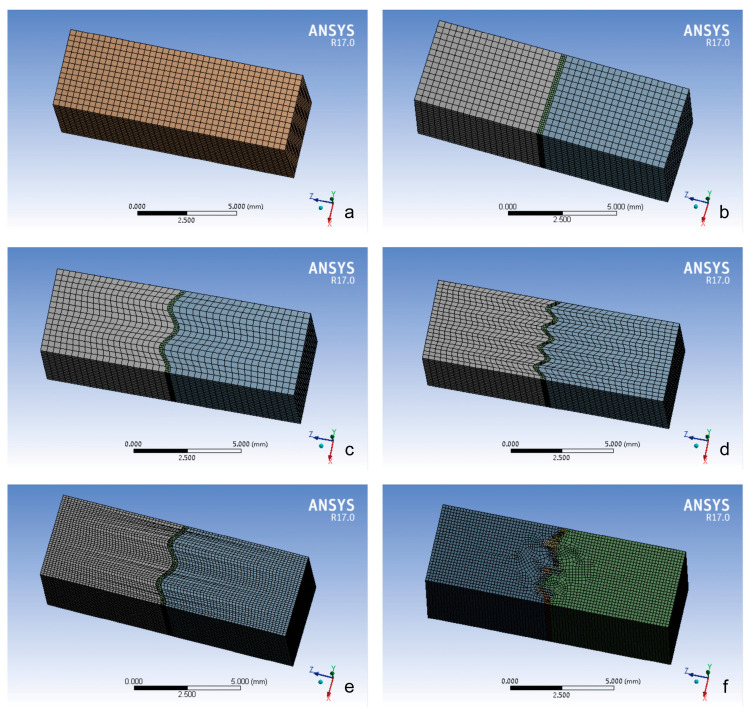
Finite element models of different cranial suture morphologies: (**a**) closed group; (**b**) straight suture group; (**c**) sine wave group; (**d**) tight sinusoidal wave group; (**e**) layered sinusoidal wave group; (**f**) layered sinusoidal wave + sutural bone group.

The idealized cranial suture was modeled based on the previous anatomical morphology of normal adult cranial sutures and literature research [3]. Specifically, suture geometry was reconstructed using micro-CT imaging data and our measurements (average thickness of 1–2 mm) of the cranial vault suture area of the human skull to ensure dimensional accuracy [14]. The models were divided into six groups with distinct morphological characteristics (Figure 1 and Figure 2):

(1) Closed group: fully ossified sutures with complete bone fusion;
(2) Straight suture group: simulating the straight line morphology of the cranial suture;
(3) Sine wave group: simulating the cranial suture as a standard sinusoidal waveform (bone–suture–bone contact surfaces are smooth with continuous mesh nodes between materials [3]; wavelength λ = 2 mm, amplitude A = 0.25 mm);
(4) Tight sinusoidal wave group: high-frequency sinusoids in which the angular velocity ω of the sine wave becomes larger, the waveform shrinks on the *X*-axis, and the wave number becomes greater compared to the sine wave group;
(5) Layered sinusoidal wave group: the structure of the cranial suture is the same as that of the standard sine wave group, but the amplitude and wavelength are different. This group is composed of two layers of sinusoidal waves, with multiple small sinusoids working together to form a large sinusoidal wave (the bone–suture–bone contact surfaces are rough);
(6) Layered sinusoidal wave + sutural bone group: hybrid structure combining sinusoidal patterns with sutural bone elements (sutural bone refers to small, irregularly shaped bone fragments or plates found within cranial sutures—the fibrous joints connecting the bones of the skull), becoming the most complex and irregular waveforms.

### 2.2. Model Construction

The three-dimensional finite element model of the cranial suture composite structure (length × width × height: 12 mm × 4 mm × 1.5 mm) was constructed using SolidWorks 2017 (Dassault Systèmes Group, Waltham, MA, USA). Cranial bone was modeled as an isotropic linear elastic material with Young’s modulus 6000 MPa and Poisson’s ratio 0.27 based on experimental data from prior studies [3,4,15,16]. The suture region was represented by two constitutive models to capture collagenous tissue anisotropy: an isotropic model for randomly oriented fibers and an orthotropic model for directional collagen matrix properties [3].

Specifically, the modeling of the sine wave group and the layered sine wave group are taken as an example. Firstly, a cuboid with a width of 0.25 mm was created, which was set to a sine wave with reference to the parameters (amplitude A = 0.25 mm, wavelength λ = 2 mm), and the sine wave model was longitudinally stretched. Then, a 12 mm × 4 mm × 1.5 mm cuboid was created and cut in the middle of the cuboid using the cranial suture model to simulate the bone–suture–bone structure of the cranial suture. The layered sinusoidal wave group model was created by superimposing primary (λ = 0.15 mm, A = 0.025 mm) and secondary (λ = 0.075 mm, A = 0.0125 mm) sinusoidal waves.

### 2.3. Meshing and Analysis

Six morphologically distinct bone–suture–bone composite models were systematically developed based on derived cranial geometry (Figure 2). Upon completion, the models were imported into ANSYS Workbench 17.0 (Ansys Corporation, Canonsburg, PA, USA) for mesh generation and material property assignment [3,4]. In addition, grid continuity at the bone–suture interface was achieved through shared nodes (ensuring no physical gaps between materials), with perfect bonding assumed between bone and suture meshes in all models. A rigorous mesh convergence study established optimal element sizes, with tetrahedral elements (0.1 mm) for cranial bone and hexahedral elements (0.05 mm) for sutural tissue. Detailed model specifications (composition, node distribution, and element sizes) are provided in Table 1.

Explicit dynamic analysis was conducted using ANSYS Workbench 17.0 with parameters including 0.1 μs time steps over a total simulation duration of 35 μs [3]. Boundary conditions consisted of a rigid constraint (x-y-z displacement fixation) at the left end [3] and free boundary conditions at the right end. Loading protocols comprised two phases. (1) Impact loading: a 50 kPa rectangular pulse (5 μs duration) applied to the left edge. (2) Tensile loading: bilateral 98 N forces (validated against craniosacral therapy standards [13]) applied to both ends. Von Mises stress distributions and total deformations were quantified for all model groups under both loading conditions.

## 3. Results

### 3.1. Von Mises Stress Changes of Different Cranial Suture Morphologies Under Impact and Tensile Loads

#### 3.1.1. Impact Loading Conditions

**Figure 3:**
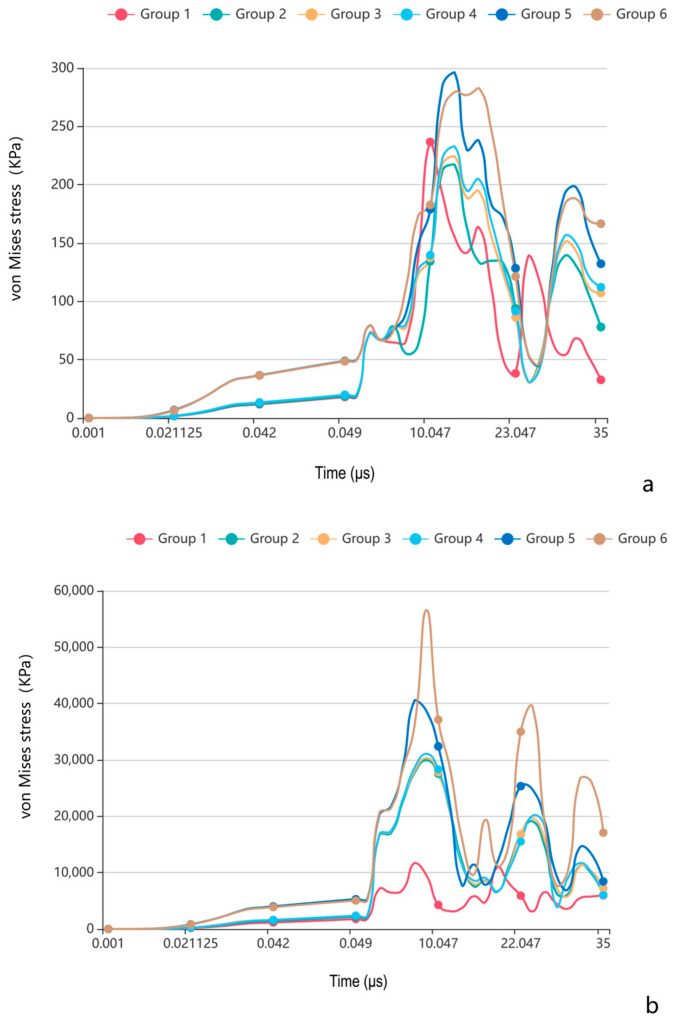
The curve of peak von Mises stress changes of different morphologies of cranial sutures with time under impact and tensile loading conditions. (**a**) Under impact loading condition; (**b**) under tensile loading condition. Group 1: closed group; Group 2: straight suture group; Group 3: sine wave group; Group 4: tight sinusoidal wave group; Group 5: layered sinusoidal wave group; Group 6: layered sinusoidal wave + sutural bone group.

The von Mises stress analysis revealed three distinct phenomena (Figure 3a):

(1) Temporal delay effect: in the cranial suture groups (excluding closed sutures), peak stress occurrence was significantly delayed compared to the closed group.
(2) Morphological equivalence: the straight suture, sine wave, and tight sinusoidal wave groups exhibited similar stress evolution trends and peak magnitudes.
(3) Hierarchical enhancement: the layered sinusoidal wave group and the layered sinusoidal wave + sutural bone group demonstrated the highest peak stresses (296.31 kPa and 282.9 kPa, respectively), surpassing all other configurations by 19–36%.

**Figure 4:**
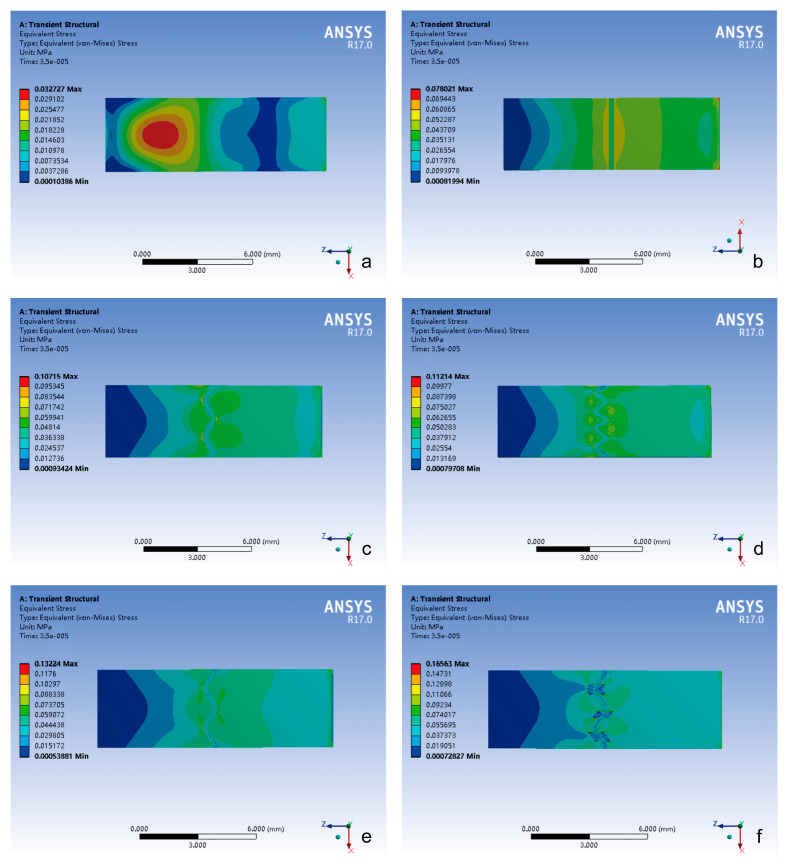
The contour plot of von Mises stress distribution in different groups under impact loading conditions. (**a**) Closed group; (**b**) straight suture group; (**c**) sine wave group; (**d**) tight sinusoidal wave group; (**e**) layered sinusoidal wave group; (**f**) layered sinusoidal wave + sutural bone group.

From the contour plot, the spatial distribution analysis indicated the following (Figure 4):

(1) The closed group exhibited stress localization primarily at the impact side or center of the composite structure.
(2) The stress of the cranial suture groups showed stress concentration on both sides of the cranial suture, with peak stress magnitude positively correlated with the complexity of the suture–bone interface.

#### 3.1.2. Tensile Loading Conditions

The peak stress temporal dynamics demonstrated the following (Figure 3b):

(1) All groups exhibited comparable peak stress timing.
(2) The layered sinusoidal wave + sutural bone group achieved the highest peak stress (56,577 kPa), followed closely by the layered sinusoidal wave group (40,611 kPa). Straight, sine, and tight sinusoidal wave groups showed nearly identical stress profiles (29,950 kPa, 30,334 kPa, 31,087 kPa), while the closed group exhibited the lowest peak stress (11,722 kPa).

**Figure 5:**
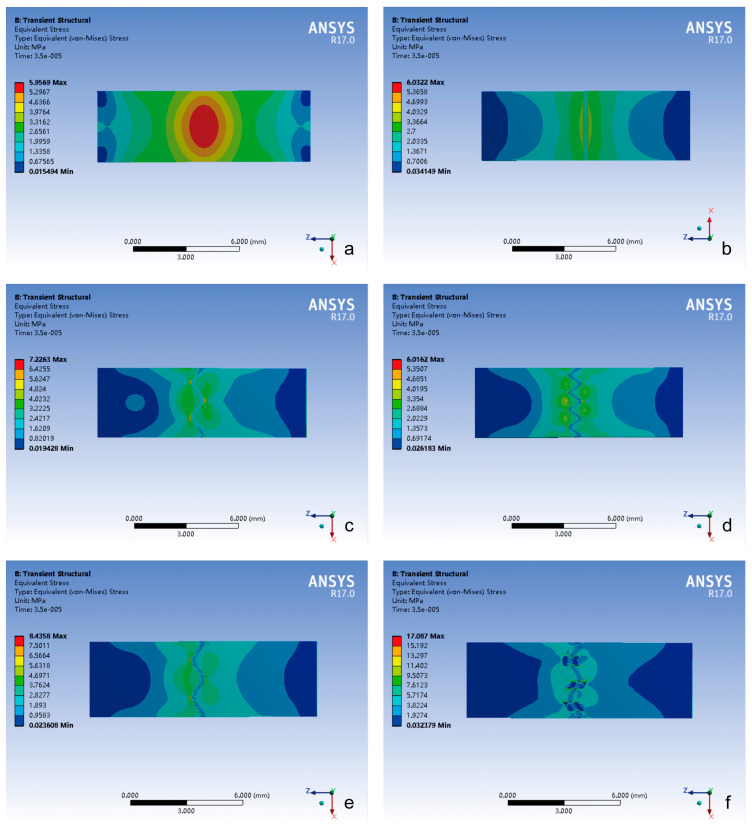
The contour plot of von Mises stress distribution in different groups under tensile loading conditions. (**a**) Closed group; (**b**) straight suture group; (**c**) sine wave group; (**d**) tight sinusoidal wave group; (**e**) layered sinusoidal wave group; (**f**) layered sinusoidal wave + sutural bone group.

The spatial distribution analysis indicated the following (Figure 5):

(1) The closed group maintained centralized stress distribution.
(2) The stress of the cranial suture groups exhibited bilateral stress localization around cranial sutures, with peak stress magnitude again positively correlated with the complexity of the suture–bone interface.

### 3.2. Total Deformation Changes of Different Cranial Suture Morphologies Under Impact and Tensile Loads

#### 3.2.1. Temporal Deformation Analysis

**Figure 6:**
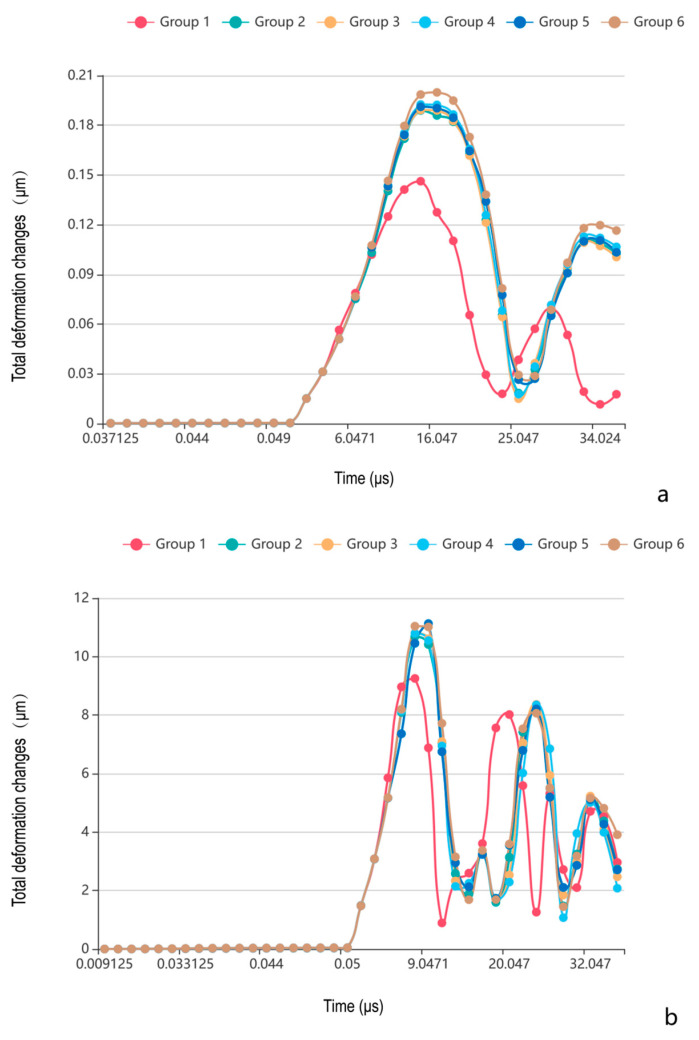
The curve of total deformation changes of different morphologies of cranial sutures with time under impact and tensile loading conditions. (**a**) Under impact loading condition; (**b**) under tensile loading condition. Group 1: closed group; Group 2: straight suture group; Group 3: sine wave group; Group 4: tight sinusoidal wave group; Group 5: layered sinusoidal wave group; Group 6: layered sinusoidal wave + sutural bone group.

The temporal deformation profiles revealed the following (Figure 6):

(1) Peak deformation timing: the cranial suture groups exhibited delayed peak deformation compared to closed groups under the impact and tensile loading conditions.
(2) Deformation magnitude hierarchy: the closed group demonstrated minimal deformation (impact: 0.14 μm; tensile: 9.25 μm); the peak value of total deformation in the cranial suture groups was significantly higher than that in the closed group, but with no inter-group differences (*p* > 0.05).
(3) Loading mode effect: the highest total deformation in all groups was only 0.2 μm under impact load and 11 μm under tensile load, and the total deformation under tensile loading is greater than that under impact loading.

#### 3.2.2. Spatial Deformation Patterns

**Figure 7:**
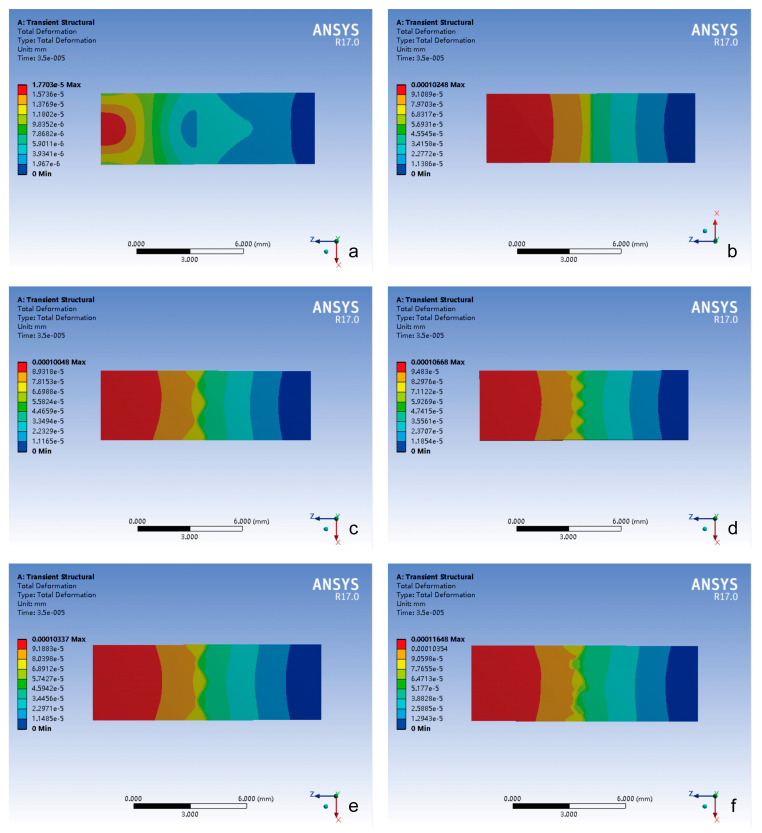
The contour plot of total deformation distribution in different groups under impact loading conditions. (**a**) Closed group; (**b**) straight suture group; (**c**) sine wave group; (**d**) tight sinusoidal wave group; (**e**) layered sinusoidal wave group; (**f**) layered sinusoidal wave + sutural bone group.

Impact Loading Conditions (Figure 7)

(1) Impact-side deformation concentration: the closed group exhibited the largest total deformation localized within the most limited impact zone, demonstrating constrained energy absorption characteristics.
(2) Attenuation gradient distribution trend: all groups showed peak total deformation concentrated on the impact side, which gradually decreased in a step-like manner.
(3) Suture-mediated deformation modulation: the presence of cranial sutures can significantly increase both peak magnitude and the spatial extent of the total deformation.
(4) Morphology–deformation correlation: the total deformation of the cranial suture was related to its morphology.

**Figure 8:**
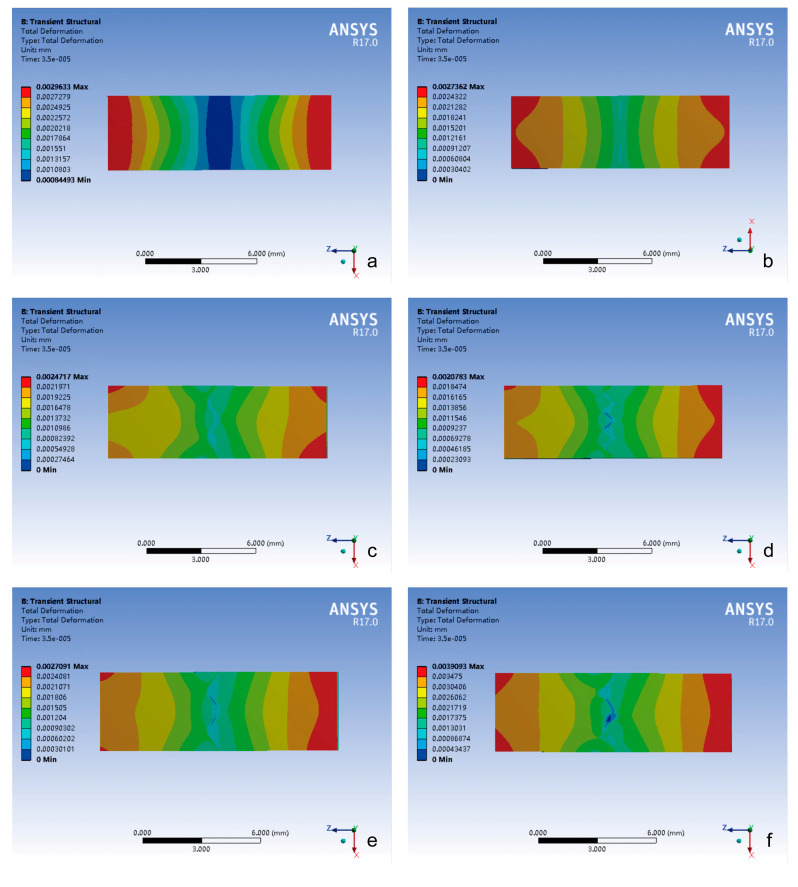
The contour plot of total deformation distribution in different groups under tensile loading conditions. (**a**) Closed group; (**b**) straight suture group; (**c**) sine wave group; (**d**) tight sinusoidal wave group; (**e**) layered sinusoidal wave group; (**f**) layered sinusoidal wave + sutural bone group.

Tensile Loading Conditions (Figure 8)

(1) Bimodal strain distribution: the total deformation of all groups exhibited symmetrical “double-peaked” distribution with maximum values at both ends and minimum values in the center.
(2) Interface complexity-driven irregularity: the distribution trend of the total deformation in the closed group maintained regular deformation distribution, whereas cranial suture groups exhibited progressive pattern irregularity proportional to suture–bone interface complexity.

## 4. Discussion

This study systematically evaluated the biomechanical behavior of cranial suture morphology under two clinically relevant loading conditions (traumatic impact and therapeutic tensile forces) by constructing six morphologically distinct finite element models that integrate biomechanical modeling with anatomical reality. Our findings reveal that suture complexity modulates stress distribution patterns and deformation dynamics, offering referential insights into cranial structural biomechanics.

### 4.1. Morphology–Environment Correlation: Mechanical Responses of Sutures Reflect Their Biomechanical Context

The constructed models, spanning from closed groups to layered sinusoidal wave + sutural bone groups (Figure 1 and Figure 2), address a critical gap in prior FEA studies that oversimplified suture geometry as idealized single-layer waveforms [7,8,9]. While Jaslow and Biewener [17] emphasized the functional significance of hierarchical cranial suture morphology, previous computational work largely ignored this layered complexity [18]. The intricate morphology of cranial sutures features interdigitating serrations marked by intersecting bony projections that form a fractal interface between adjacent bones [17,19]. These interlocking geometries enhance shear resistance by distributing mechanical loads across staggered suture–bone interfaces, effectively counteracting multidirectional forces [17,19]. Similarly, layered sutural architectures amplify energy absorption capacity through nested waveform hierarchies, optimizing strain dissipation via increased surface area and interfacial complexity [17].

The peak von Mises stresses observed in these complex configurations (Figure 3, Figure 4 and Figure 5) positively correlated with the complexity of the suture–bone interface, underscoring how morphological intricacy redistributes mechanical loads. This aligns with anatomical observations of rough, interlocking suture surfaces in unclosed cranial sutures [10,11], suggesting evolutionary optimization for stress dissipation. In our previous anatomical study, we found that the closure rate of the extracranial suture in Chinese adults was 31.93%, and the closure rate of the intracranial suture was higher than that of the extracranial suture; in the unclosed cranial sutures, the bony surfaces on both sides of the bone–suture–bone composite structures were rough and embedded with each other, which made it difficult to move.

### 4.2. Suture Function Under Contrasting Loading Regimes

The bone–suture–bone structure is a composite mechanical structure [20], which plays a role in bearing and transferring loads and absorbing energy to some extent [17,19]. Under impact loading simulating traumatic forces, the cranial suture groups’ peak stress occurrence was significantly delayed compared to the closed group, the layered sinusoidal wave group, and the layered sinusoidal wave + sutural bone group delayed peak stress occurrence by 19–36% compared to closed configurations (Figure 3a). This temporal delay phenomenon aligns with viscoelastic wave impedance theory [3,21] and underscores sutures’ role as biomechanical dampers, where morphological complexity prolongs energy dissipation through staggered interfacial load transfer [19,22]. In addition, the spatial stress shift from localized impact zones in closed sutures to bilateral suture–bone interfaces in open morphologies (Figure 4) highlights how sutures diffuse energy across broader anatomical regions [17,22]. The observed bilateral stress concentration around sutures (Figure 4 and Figure 5) demonstrates a direct correlation between interface complexity and mechanical performance, validating hierarchical structures’ advantage in stress dissipation [17,19,22].

Total deformation analysis revealed two distinct loading-dependent regimes with critical implications for cranial biomechanics and clinical safety (Figure 6, Figure 7 and Figure 8). Under impact loading, cranial sutures exhibited localized microscale deformations (<0.2 μm) at suture margins (Figure 6 and Figure 7), demonstrating evolved viscoelastic relaxation mechanisms that enable energy dissipation without catastrophic structural failure. Conversely, tensile loading mimicking CST forces revealed suture morphology’s limited influence on deformation magnitude (*p* > 0.05), with all sutured groups exhibiting micron-scale displacements (≤11 μm, Figure 6). This response reveals fundamental mechanical differentiation; while impact loads exploit suture geometry for targeted energy attenuation, tensile loads test structural tolerance limits. Notably, the observed microscale deformations under craniosacral therapy loads (98 N) [13] align with physiological force ranges documented in animal studies [19,23], confirming that recommended manipulation forces do not induce pathological cranial suture displacement (≥1 mm) [13].

### 4.3. Clinical and Evolutionary Implications

The complexity of cranial suture morphology is difficult to characterize, as the mechanical behavior integrates critical biological functions across ontogeny and phylogeny [24,25]. Beyond impact protection, our results contextualize suture mechanics within broader craniofacial biology. (1) Developmental plasticity: the correlation between interfacial complexity and stress magnitude (Figure 3, Figure 4 and Figure 5) supports hypotheses linking mechanical strain to suture morphogenesis. These factors include innovation at evolution, novel biomechanical relationships with ecological changes, stress-induced mechanical strain, growth and developmental patterns, and responses to environmental stresses [26,27,28,29]. Compressive/tensile forces during mastication or growth may drive interdigitation to optimize load distribution [30,31,32,33]. (2) Pathological ossification: the cranial suture serves as the main developmental site for intramembranous osteogenesis, which determines the morphology and size of the cranium [2,34,35], but the closed group’s restricted deformation (Figure 6) illustrates the biomechanical cost of premature fusion, potentially exacerbating trauma vulnerability [24,36]. (3) CST biomechanics: the minimal deformation under 98 N tensile loads (validated against CST protocols) suggests that reported clinical effects [19] likely arise from non-mechanical mechanisms, such as neuromodulation or placebo responses.

## 5. Conclusions

By integrating morphometric diversity into finite element frameworks, this study establishes cranial sutures as dynamic stress modulators whose biomechanical efficacy depends on interfacial complexity rather than gross morphology. The findings provide mechanistic explanations for suture evolution, pathological vulnerability, and clinical intervention limitations, offering a quantitative foundation for future research in cranial biomechanics and therapeutic innovation.

## 6. Study Limitations and Future Directions

While our finite element models represent an advance in cranial suture biomechanical analysis through geometrically informed approximations, several limitations warrant consideration to guide future research. The sinusoidal approximations, though anatomically informed [10,11], cannot fully replicate the stochastic fractal patterns observed in vivo. Although bone tissue was modeled as homogeneous isotropic material based on established protocols, this assumption neglects the complex anisotropy arising from osteonal microstructure and regional density variations [24,37]. The controversial yet pragmatically employed von Mises criterion, while validated for axial loading scenarios in homogeneous bone regions [1,24], requires cautious interpretation given the lack of experimental confirmation for suture–bone interfacial stresses. Future work should incorporate micro-CT-derived morphologies and viscoelastic/poroelastic suture properties [9,33]. Additionally, validating these findings against in vitro mechanical testing of cadaveric specimens would strengthen translational relevance.

## Figures and Tables

**Table 1:** Material properties and the number and size of the elements for each model.

Model Group	Component	Nodes	Elements	Element Size (mm)
Closed Group	Cranial Bone	42,657	9520	Bone: 0.3
Straight Suture Group	Bone	21,717	4760	Bone: 0.15
	Suture	30,997	6000	Suture: 0.1
Sine Wave Group	Bone	23,190	5100	Bone: 0.15
	Suture	35,563	6900	Suture: 0.1
Tight Sinusoidal Wave Group	Bone	32,028	7140	Bone: 0.15
	Suture	45,456	8850	Suture: 0.1
Layered Sinusoidal Wave Group	Bone (Side 1)	236,453	55,760	Bone: 0.3
	Bone (Side 2)	230,780	54,400	Bone: 0.3
	Suture	78,325	48,854	Suture: 0.1
Layered Sinusoidal & Sutural Bone Group	Bone (Side 1)	162,296	37,570	Bone: 0.15
	Bone (Side 2)	174,076	40,426	Bone: 0.15
	Sutural Bone 1	5884–7406	1050–1350	Suture: 0.1
	Sutural Bone 2	5884–7406	1050–1350	Suture: 0.1
	Sutural Bone 3	5884–7406	1050–1350	Suture: 0.1
	Remaining Suture Segments	~6000	~1100	Suture: 0.1

Closed Group: Single cranial bone component without sutures. Straight/Sine/Tight Sinusoidal Groups: Three-part models (two bones + suture). Bone element size: 0.15 mm (all except layered sinusoidal wave group). Suture element size: 0.1 mm for all. Layered Sinusoidal Wave Group: Bilateral bone components with large node/element counts. Layered Sinusoidal & Sutural Bone Group: Complex 15-part model with heterogeneous sutural bone regions.

## Data Availability

The datasets used and analyzed during the current study are available from the corresponding authors upon reasonable request.

## References

[1] White H.E., Clavel J., Tucker A.S., Goswami A. (2020). A comparison of metrics for quantifying cranial suture complexity. J. R. Soc. Interface.

[2] White H.E., Goswami A., Tucker A.S. (2021). The Intertwined Evolution and Development of Sutures and Cranial Morphology. Front. Cell Dev. Biol..

[3] Zhang Z.Q., Yang J.L. (2015). Biomechanical Dynamics of Cranial Sutures during Simulated Impulsive Loading. Appl. Bionics Biomech..

[4] Remesz R., Khurelbaatar T., Grotski M., Popowics T., Rafferty K., Herring S.W., Addison O., Doschak M.R., Romanyk D.L. (2022). Cranial suture morphometry and mechanical response to loading: 2D vs. 3D assumptions and characterization. Biomech. Model. Mechanobiol..

[5] Sharp A.C., Dutel H., Watson P.J., Gröning F., Crumpton N., Fagan M.J., Evans S.E. (2023). Assessment of the mechanical role of cranial sutures in the mammalian skull: Computational biomechanical modelling of the rat skull. J. Morphol..

[6] Wang Q., Smith A.L., Strait D.S., Wright B.W., Richmond B.G., Grosse I.R., Byron C.D., Zapata U. (2010). The global impact of sutures assessed in a finite element model of a macaque cranium. Anat. Rec..

[7] Rayfield E.J. (2005). Using finite-element analysis to investigate suture morphology: A case study using large carnivorous dinosaurs. Anat. Rec. A Discov. Mol. Cell Evol. Biol..

[8] Jasinoski S.C., Reddy B.D., Louw K.K., Chinsamy A. (2010). Mechanics of cranial sutures using the finite element method. J. Biomech..

[9] Maloul A., Fialkov J., Wagner D., Whyne C.M. (2014). Characterization of craniofacial sutures using the finite element method. J. Biomech..

[10] Li J., Chen Z., Zhong W., Yang H., Li Y. (2022). A study of 285 cases of cranial vault suture closure in Chinese adults. Surg. Radiol. Anat..

[11] Li J.H., Chen Z.J., Zhong W.X., Yang H., Liu D., Li Y.K. (2023). Anatomical characteristics and significance of the metopism and Wormian bones in dry adult-Chinese skulls. Folia Morphol..

[12] Rogers J.S., Witt P.L. (1997). The controversy of cranial bone motion. J. Orthop. Sports Phys. Ther..

[13] Downey P.A., Barbano T., Kapur-Wadhwa R., Sciote J.J., Siegel M.I., Mooney M.P. (2006). Craniosacral therapy: The effects of cranial manipulation on intracranial pressure and cranial bone movement. J. Orthop. Sports Phys. Ther..

[14] Corega C., Vaida L., Băciuţ M., Serbănescu A., Palaghiţă-Banias L. (2010). Three-dimensional cranial suture morphology analysis. Rom. J. Morphol. Embryol..

[15] Ashman R.B., Van Buskirk W.C. (1987). The elastic properties of a human mandible. Adv. Dent. Res..

[16] Coats B., Margulies S.S. (2006). Material properties of human infant skull and suture at high rates. J. Neurotrauma.

[17] Jaslow C.R. (1990). Mechanical properties of cranial sutures. J. Biomech..

[18] Zollikofer C.P., Weissmann J.D. (2011). A bidirectional interface growth model for cranial interosseous suture morphogenesis. J. Anat..

[19] Herring S.W., Mucci R.J. (1991). In vivo strain in cranial sutures: The zygomatic arch. J. Morphol..

[20] Tanaka E., Miyawaki Y., del Pozo R., Tanne K. (2000). Changes in the biomechanical properties of the rat interparietal suture incident to continuous tensile force application. Arch. Oral. Biol..

[21] Ox R.H. (1968). Wave propagation through a newtonian fluid contained within a thick-walled, viscoelastic tube. Biophys. J..

[22] Herring S.W., Ochareon P. (2005). Bone–special problems of the craniofacial region. Orthod. Craniofacial Res..

[23] Losken H.W., Mooney M.P., Zoldos J., Tschakaloff A., Burrows A.M., Smith T.D., Cano G., Arnott R., Sherwood C., Dechant J. (1999). Coronal suture response to distraction osteogenesis in rabbits with delayed-onset craniosynostosis. J. Craniofacial Surg..

[24] Ruengdit S., Troy Case D., Mahakkanukrauh P. (2020). Cranial suture closure as an age indicator: A review. Forensic Sci. Int..

[25] Di Ieva A., Bruner E., Davidson J., Pisano P., Haider T., Stone S.S., Cusimano M.D., Tschabitscher M., Grizzi F. (2013). Cranial sutures: A multidisciplinary review. Childs Nerv. Syst..

[26] Monteiro L.R., Lessa L.G. (2000). Comparative analysis of cranial suture complexity in the genus Caiman (Crocodylia, Alligatoridae). Braz. J. Biol..

[27] Henderson J.H., Chang L.Y., Song H.M., Longaker M.T., Carter D.R. (2005). Age-dependent properties and quasi-static strain in the rat sagittal suture. J. Biomech..

[28] Nicolay C.W., Vaders M.J. (2006). Cranial suture complexity in white-tailed deer (*Odocoileus virginianus*). J. Morphol..

[29] Wood C. (2015). The age-related emergence of cranial morphological variation. Forensic Sci. Int..

[30] Herring S.W., Teng S. (2000). Strain in the braincase and its sutures during function. Am. J. Phys. Anthropol..

[31] Byron C.D., Borke J., Yu J., Pashley D., Wingard C.J., Hamrick M. (2004). Effects of increased muscle mass on mouse sagittal suture morphology and mechanics. Anat. Rec. A Discov. Mol. Cell Evol. Biol..

[32] Buezas G., Becerra F., Vassallo A. (2017). Cranial suture complexity in caviomorph rodents (Rodentia; Ctenohystrica). J. Morphol..

[33] Byron C., Segreti M., Hawkinson K., Herman K., Patel S. (2018). Dietary material properties shape cranial suture morphology in the mouse calvarium. J. Anat..

[34] Opperman L.A. (2000). Cranial sutures as intramembranous bone growth sites. Dev. Dyn..

[35] Geiger M., Haussman S. (2016). Cranial Suture Closure in Domestic Dog Breeds and Its Relationships to Skull Morphology. Anat. Rec..

[36] Hukki J., Saarinen P., Kangasniemi M. (2008). Single suture craniosynostosis: Diagnosis and imaging. Front. Oral. Biol..

[37] Curtis N., Jones M.E., Evans S.E., O’Higgins P., Fagan M.J. (2013). Cranial sutures work collectively to distribute strain throughout the reptile skull. J. R. Soc. Interface.

